# Novel Pathogenic *PRSS1* Variant p.Glu190Lys in a Case of Chronic Pancreatitis

**DOI:** 10.3389/fgene.2019.00046

**Published:** 2019-02-06

**Authors:** Zsanett Jancsó, Grzegorz Oracz, Aleksandra Anna Kujko, Eliwira Kolodziejczyk, Evette S. Radisky, Agnieszka Magdalena Rygiel, Miklós Sahin-Tóth

**Affiliations:** ^1^Center for Exocrine Disorders, Department of Molecular and Cell Biology, Boston University Henry M. Goldman School of Dental Medicine, Boston, MA, United States; ^2^Department of Gastroenterology, Hepatology, Feeding Disorders and Pediatrics, Children’s Memorial Health Institute, Warsaw, Poland; ^3^Department of Medical Genetics, Institute of Mother and Child, Warsaw, Poland; ^4^Department of Cancer Biology, Mayo Clinic Cancer Center, Jacksonville, FL, United States

**Keywords:** pancreas, hereditary pancreatitis, autoactivation, trypsinogen mutant, digestive protease

## Abstract

Mutations in the *PRSS1* (serine protease 1) gene encoding human cationic trypsinogen cause hereditary pancreatitis or may be associated with sporadic chronic pancreatitis. The mutations exert their pathogenic effect either by increasing intra-pancreatic trypsinogen activation (trypsin pathway) or by causing proenzyme misfolding and endoplasmic reticulum stress (misfolding pathway). Here we report a novel heterozygous c.568G>A (p.Glu190Lys) variant identified in a case with chronic pancreatitis. The parents of the index patient had no history of pancreatitis but were unavailable for genetic testing. Functional characterization revealed 2.5-fold increased autoactivation of the mutant trypsinogen relative to wild type. Unlike many other clinically relevant *PRSS1* mutations, p.Glu190Lys did not alter the chymotrypsin C (CTRC)-dependent degradation of trypsinogen nor did it increase CTRC-mediated processing of the trypsinogen activation peptide. Cellular secretion of the mutant protein was unchanged indicating normal folding behavior. Based on the genetic and functional evidence, we classify the p.Glu190Lys *PRSS1* variant as likely pathogenic, which stimulates autoactivation of cationic trypsinogen independently of CTRC.

## Introduction

Mutations in the *PRSS1* (serine protease 1) gene encoding human cationic trypsinogen have been found in families with autosomal dominant hereditary pancreatitis and in sporadic cases without a family history (Whitcomb et al., 1996; Németh and Sahin-Tóth, 2014). Studies spanning almost two decades revealed that *PRSS1* mutations cause pancreatitis via two different mechanisms; the trypsin-dependent and the misfolding dependent pathways (Hegyi and Sahin-Tóth, 2017; Sahin-Tóth, 2017). The majority of clinically relevant mutations exert their effect by increasing intra-pancreatic autoactivation of cationic trypsinogen resulting in elevated levels of harmful trypsin activity (Hegyi and Sahin-Tóth, 2017). Most of these mutations tend to associate with hereditary pancreatitis. A smaller number of mutations cause misfolding of cationic trypsinogen, which elicits endoplasmic reticulum stress and damages acinar cells (Sahin-Tóth, 2017). Misfolding mutants are more often found in sporadic cases although they have been also observed in families with hereditary pancreatitis (Németh et al., 2017a). *PRSS1* mutations that act through the trypsin-dependent pathway can be further subdivided according to their molecular mechanisms. While the common biochemical phenotype is increased autoactivation of trypsinogen, this effect may be achieved in a number of different ways. The clinically most frequent mutation p.Arg122His blocks chymotrypsin C (CTRC)-dependent degradation of cationic trypsinogen and thereby increases autoactivation and accumulation of trypsin (Szabó and Sahin-Tóth, 2012). Similarly, mutations p.Asn29Ile, p.Asn29Thr, p.Val39Ala and p.Arg122Cys decrease or block CTRC-dependent trypsinogen degradation (Szabó and Sahin-Tóth, 2012). CTRC is a digestive chymotrypsin isoform, which controls activation of human cationic trypsinogen via regulatory nick sites. Thus, cleavage after Leu81 in the calcium-binding loop facilitates trypsinogen degradation, a process that also requires an autolytic cleavage by trypsin after Arg122 (Szmola and Sahin-Tóth, 2007; Szabó and Sahin-Tóth, 2012). Furthermore, CTRC cleaves the trypsinogen activation peptide after Phe18 and shortens it by three amino acids. Trypsinogen with a CTRC-processed activation peptide exhibits increased autoactivation (Nemoda and Sahin-Tóth, 2006). While CTRC seems to have two opposing effects on trypsinogen activation, under normal circumstances degradation dominates and activation peptide processing is not significant. However, *PRSS1* mutations that increase processing of the activation peptide (p.Ala16Val, p.Pro17Thr, and p.Asn29Ile) shift this balance and result in increased autoactivation with elevated trypsin levels (Nemoda and Sahin-Tóth, 2006; Szabó and Sahin-Tóth, 2012; Németh et al., 2017b). Finally, a subset of mutations that affect the activation peptide (p.Asp19Ala, pAsp20Ala, p.Asp22Gly, and p.Lys23Arg) directly stimulate autoactivation in a manner that is independent of CTRC function (Geisz et al., 2013).

Here we report the novel *PRSS1* c.568G>A (p.Glu190Lys) mutation identified in a case of chronic pancreatitis. Functional studies revealed that the mutation increases autoactivation of cationic trypsinogen and, therefore, should be considered likely pathogenic associated with the trypsin-dependent pathological pathway.

## Materials and Methods

### Nomenclature

Amino-acid residues in human cationic trypsinogen were numbered starting with the initiator methionine of the primary translation product, according to the recommendations of the Human Genome Variation Society. The reference sequence used was NM_002769.4.

### Genotyping

This study was carried out in accordance with the Declaration of Helsinki. The study protocol was approved by the Committee on Bioethics at the Children’s Memorial Health Institute, Warsaw, Poland. The parents of the minor index patient gave written informed consent for genetic analysis in 2002. More recently, written informed consent was also obtained from the now adult index patient for the publication of this case report. Genetic analysis was performed at the Institute of Mother and Child, Warsaw, Poland. Genomic DNA was isolated from peripheral blood mononuclear cells using GenomicMaxi AX (A&A Biotechnology, Gdynia, Poland). DNA was amplified by PCR and sequenced using the Sanger method. All exons and exon–intron junctions of *PRSS1, SPINK1*, and *CPA1*, exons 2–7 of *CTRC* and exons 4 and 9–11 of *CFTR* were sequenced. The presence of the dele2,3(21 kb) mutation in *CFTR* was investigated by PCR. Large unbalanced rearrangements in *PRSS1* were excluded by Multiplex Ligation-dependent Probe Amplification (SALSA MLPA P242 pancreatitis; MRC Holland, Amsterdam, Netherlands).

### Structure Models

The structure of human cationic trypsin was rendered from the coordinates of Protein Data Bank entry 2RA3 (Salameh et al., 2008), and the p.Glu190Lys mutation was incorporated using the PyMOL v. 1.6 software (Schrödinger). Electrostatic surface potentials were calculated using the Bluues server v. 2.0 which employs a generalized Born model (Fogolari et al., 2012), and were rendered in PyMOL.

### Plasmid Construction and Mutagenesis

Construction of the pTrapT7 intein-PRSS1, and pcDNA3.1(–) PRSS1 expression plasmids were reported previously (Király et al., 2006; Nemoda and Sahin-Tóth, 2006). Missense mutation c.568G>A (p.Glu190Lys) was introduced by overlap extension PCR mutagenesis.

### Expression and Purification of Recombinant PRSS1 and CTRC

Human cationic trypsinogen was expressed in the aminopeptidase P deficient LG-3 *Escherichia coli* strain as an intein-trypsinogen fusion construct (Király et al., 2006). During expression, the mini-intein moiety undergoes spontaneous self-splicing and liberates the trypsinogen with a homogeneous, authentic N terminus. *In vitro* refolding and purification of trypsinogen on an ecotin affinity column was performed as described previously (Király et al., 2011). Concentrations of trypsinogen solutions were determined from the UV absorbance at 280 nm using the extinction coefficient 37,525 M^-1^ cm^-1^. Human CTRC carrying a C-terminal 10His affinity tag was expressed in HEK 293T cells, purified, activated by trypsin and quantitated by active site titration as described in Szabó and Sahin-Tóth (2012).

### Trypsinogen Autoactivation

Experiments to characterize autoactivation of wild-type and p.Glu190Lys mutant cationic trypsinogen were carried out at pH 8.0 in 1 mM CaCl_2_, in the absence or presence of human CTRC, as reported previously (Szabó and Sahin-Tóth, 2012). Trypsin activity was expressed as percent of maximal activity, which was determined by autoactivation in 10 mM CaCl_2_.

### Kinetic Analysis

Michaelis–Menten parameters of trypsin activity were measured at 22°C in 0.1 M Tris–HCl (pH 8.0), 1 mM CaCl_2_, and 0.05% Tween 20. The concentration of trypsin was 5 nM and the concentration of the *N*-CBZ-Gly-Pro-Arg-*p*-nitroanilide peptide substrate varied between 1 and 150 μM. Initial velocities of substrate cleavage were plotted as a function of substrate concentration and data points were fitted with the Michaelis–Menten hyperbolic equation.

### Cell Culture and Transfection

Human embryonic kidney (HEK) 293T cells were cultured and transfected with 4 μg pcDNA3.1(–) PRSS1 expression plasmid as described previously (Balázs et al., 2016). After overnight incubation at 37°C, cells were rinsed and overlaid with 2 mL OptiMEM medium. Media and cells were collected 48 h after this medium change.

### Measurement of Trypsinogen Secretion

Secreted trypsinogen in the conditioned medium was activated by human enteropeptidase and trypsin activity was measured with the *N*-CBZ-Gly-Pro-Arg-*p*-nitroanilide substrate, as reported previously (Balázs et al., 2016). For SDS-PAGE analysis, 200 μL conditioned medium was precipitated with 10% trichloroacetic acid (final concentration), resuspended in reducing Laemmli sample buffer, electrophoresed on 15% minigels and stained with Coomassie Blue.

## Results

### Case Report

An 11-year-old girl with prehepatic portal hypertension due to portal vein thrombosis was referred to the Children’s Memorial Health Institute, Warsaw, Poland, following two episodes of acute pancreatitis. The age of disease onset was 9.7 years. Abdominal ultrasound revealed chronic pancreatitis with a heterogeneous pancreas and a dilated pancreatic duct. Risk factors of pancreatitis such as injury, anatomical anomalies, toxic-metabolic disorders and biliary disease were excluded. Genetic testing revealed the presence of a heterozygous c.568G>A (p.Glu190Lys) variant in *PRSS1*. No other pathogenic variants were identified in the susceptibility genes tested in this patient. The parents of the index patient had no history of pancreatitis but were unavailable for genetic testing The p.Glu190Lys variant is not listed in the 1000 genomes, dbSNP, genomic GNomad (version 2.0.2), ClinVar and HGMD databases.

### Modeling the Effects of Mutation p.Glu190Lys

**FIGURE 1:**
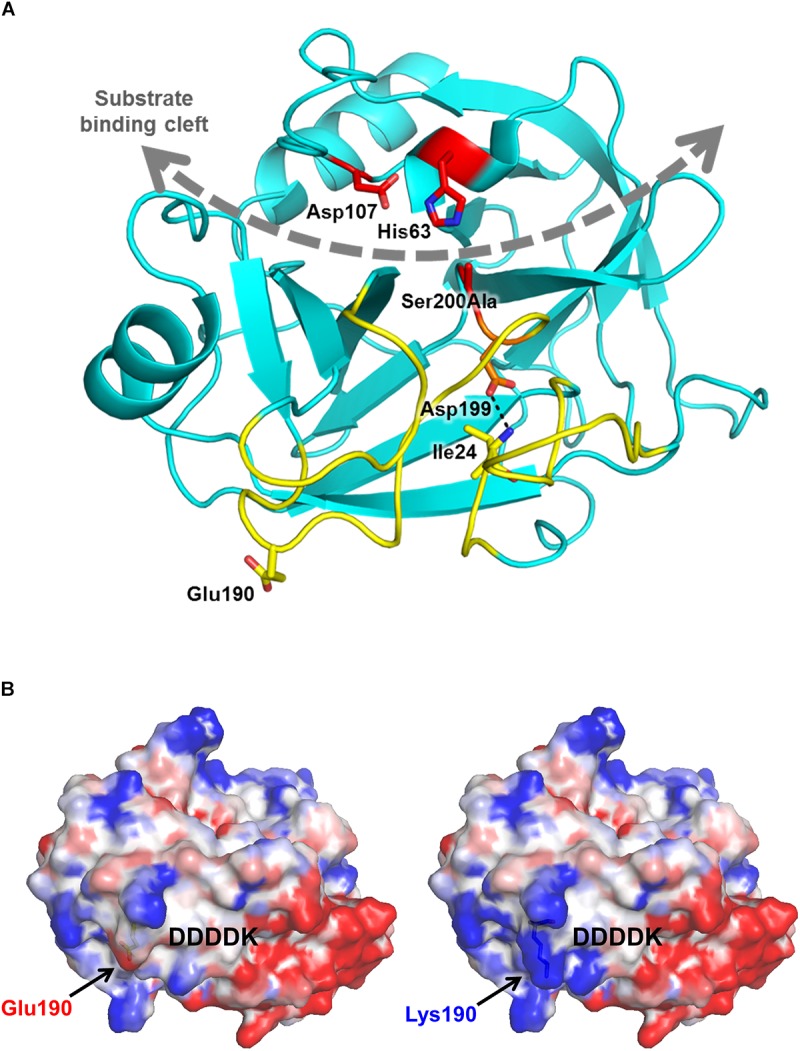
Mutation p.Glu190Lys alters the electrostatic surface potential of human cationic trypsinogen. **(A)** Ribbon diagram of human cationic trypsin (Protein Data Bank ID 2RA3) shows the position of Glu190 (yellow sticks) relative to the substrate binding cleft (gray curved dashed arrow) and active site catalytic triad of Ser200 (here mutated to Ala), His63, and Asp107 (red sticks). Glu190 lies within the activation domain (yellow ribbons), comprised of the N-terminus and three surface loops that are unstructured in trypsinogen. Upon proteolytic activation to trypsin by cleavage of the Lys23-Ile24 bond, the new N-terminus Ile24 forms a salt bridge (dotted black line) with Asp199 to stabilize the mature trypsin fold. **(B)** Calculated electrostatic surface potentials for wild-type (left) and p.Glu190Lys human cationic trypsin (right) show localized regions of positive charge (blue) and negative charge (red) in the activation domain, flanking the position from which the activation peptide of trypsinogen (indicated by the DDDDK motif) would extend prior to its proteolytic removal. The p.Glu190Lys mutation confers a greater intensity of positive charge to a basic patch to the left of the activation peptide.

The *in silico* prediction tools MutationTaster, SIFT and PolyPhen-2 classify the variant as damaging. Molecular modeling based on the known crystal structure of human cationic trypsin (Salameh et al., 2008) reveals that the mutation is located on the surface, far from the substrate binding cleft and catalytic site (Figure 1A). The replacement of the negatively charged Glu190 side chain with a positive Lys side chain changes the surface potential of trypsinogen, intensifying the positive charge concentration of a basic patch (Figure 1B). It is possible that this alteration in surface potential contributes to increased charge-dependent macroscopic attraction between trypsin and trypsinogen, accelerating autoactivation. A similar mechanism was described for the specific recognition of cationic trypsinogen by CTRC (Batra et al., 2013), and distribution of surface charge has likewise been reported previously as a determinant of trypsinogen autoactivation behavior (Buettner et al., 2014). Alternatively, the basic surface patch may interact with the adjacent activation peptide and improve its recognition as a substrate for trypsin, resulting in increased autoactivation. The trypsinogen activation peptide features a negatively charged tetra-aspartate motif which may ion-pair with the newly created Lys190 and help to orient the activation peptide in the extended conformation required for proteolysis (Figure 1B). Taken together, observations from modeling predict increased autoactivation of trypsinogen and unaltered catalytic activity of trypsin.

### Functional Analysis of Mutation p.Glu190Lys

To validate our predictions experimentally, we produced wild-type and mutant cationic trypsinogen recombinantly and compared catalytic activity, autoactivation and the effect of CTRC on the two preparations, as detailed below. We also transfected cells with the wild-type and mutant expression constructs and evaluated cellular secretion.

### Catalytic Activity of p.Glu190Lys Cationic Trypsin

Kinetic parameters of wild-type and mutant enzymes were comparable on the small peptide substrate N-CBZ-Gly-Pro-Arg-*p*-nitroanilide (Table 1).

**Table 1:** Kinetic parameters of wild-type and p.Glu190Lys mutant trypsin on the *N*-CBZ-Gly-Pro-Arg-*p*-nitroanilide peptide substrate measured at 22°C in 0.1 M Tris–HCl (pH 8.0), 1 mM CaCl_2_, and 0.05% Tween 20 (final concentrations).

	*k*_cat_^(s-1^)	*K*_M_ (μM)	*k*_cat_/*K*_M_ (M^-1^ s^-1^)
Wild type	39.5 ± 1.4	18.9 ± 0.8	2.1 × 10^6^
p.Glu190Lys	36.5 ± 1.3	21.8 ± 0.9	1.7 × 10^6^


### Autoactivation of p.Glu190Lys Cationic Trypsinogen

**FIGURE 2:**
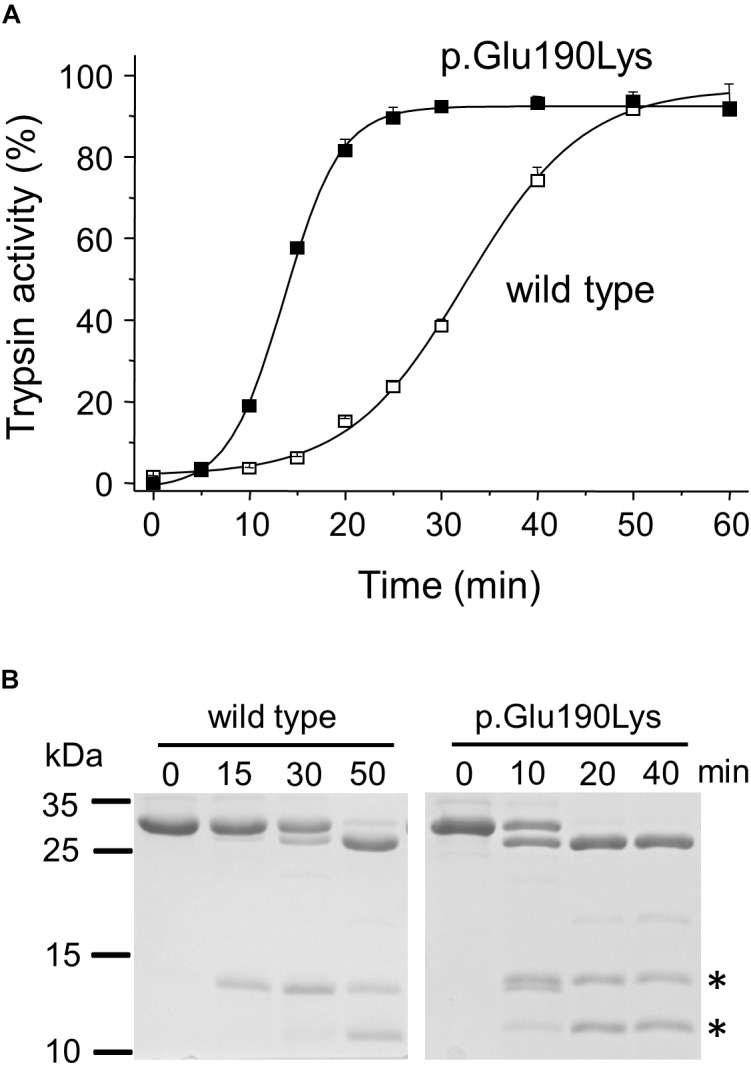
Functional effects of mutation p.Glu190Lys in PRSS1. **(A,B)** Effect of mutation p.Glu190Lys on the autoactivation of human cationic trypsinogen. Wild-type and mutant human cationic trypsinogen (1 μM) were incubated with 10 nM initial trypsin in 0.1 M Tris–HCl (pH 8.0), 1 mM CaCl_2_, and 0.05% Tween 20 (final concentrations) at 37°C. **(A)** At the indicated times, 5 μL aliquots were removed, and trypsin activity was measured with 150 μM *N*-Suc-Ala-Ala-Pro-Lys-*p*-nitroanilide substrate. Trypsin activity was expressed as percent of maximal activity, which was determined in a separate autoactivation reaction in 10 mM CaCl_2_. Data points represent mean ± SD (*n* = 3). **(B)** Autoactivation was also followed by SDS-PAGE. At the indicated times, 150 μL aliquots were precipitated with 10% trichloroacetic acid (final concentration), electrophoresed on 15% SDS-PAGE gels, and stained with Coomassie Blue. A representative gel from two experiments is shown. The asterisks indicate the proteolytic fragments generated by autolytic cleavage at the Arg122-Val123 peptide bond.

When autoactivation was measured at pH 8.0 in 1 mM calcium, the p.Glu190Lys mutant trypsinogen autoactivated about 2.5-fold faster than wild type (Figure 2A). SDS-PAGE analysis of the autoactivation reaction confirmed that the p.Glu190Lys trypsinogen band was converted to the faster-migrating trypsin band at a more rapid rate, relative to the wild-type trypsinogen band. Although mutation p.Glu190Lys creates a new trypsin-sensitive peptide bond, we did not observe cleavage at this site. Prominent proteolytic fragments corresponded to those resulting from cleavage of the Arg122-Val123 peptide bond (Figure 2B).

### Autoactivation of p.Glu190Lys Cationic Trypsinogen in the Presence of CTRC

**FIGURE 3:**
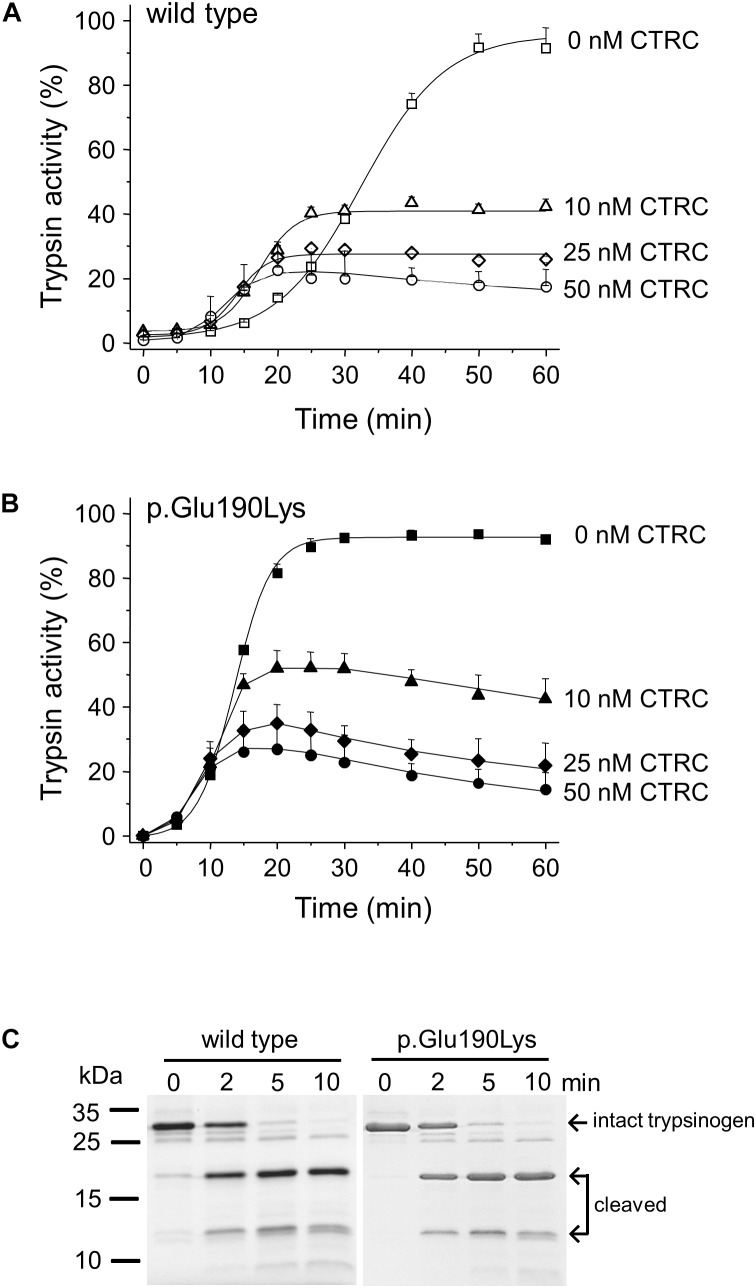
Interaction of cationic trypsinogen mutant p.Glu190Lys with chymotrypsin C (CTRC). **(A,B)** Effect of mutation p.Glu190Lys on the autoactivation of human cationic trypsinogen in the presence of CTRC. Wild-type **(A)** and mutant **(B)** trypsinogen (1 μM) were incubated at 37°C in the absence or presence of the indicated concentrations of human CTRC and 10 nM cationic trypsin in 0.1 M Tris–HCl (pH 8.0), 1 mM CaCl_2_, and 0.05% Tween 20 (final concentrations). At the indicated times, 5 μL aliquots were removed, and trypsin activity was measured with 150 μM *N*-Suc-Ala-Ala-Pro-Lys-*p*-nitroanilide substrate. Trypsin activity was expressed as percent of the maximal activity measured in the absence of CTRC in 10 mM CaCl_2_. Data points represent mean ± SD (*n* = 3). **(C)** Effect of mutation p.Glu190Lys on the cleavage of the Leu81-Glu82 peptide bond in trypsinogen by CTRC. Wild-type and mutant human cationic trypsinogen (1 μM) were incubated at 37°C with 25 nM human CTRC in 0.1 M Tris–HCl (pH 8.0) (final concentrations) in the presence of SPINK1. At the indicated times, 150 μL aliquots were precipitated with 10% trichloroacetic acid, electrophoresed on 15% SDS-PAGE gels, and stained with Coomassie Blue. Representative gel from two experiments is shown.

Degradation of wild-type and p.Glu190Lys trypsinogen by CTRC during autoactivation was comparable and final trypsin levels were similarly suppressed (Figure 3A,B). However, due to its intrinsically faster autoactivation, mutant p.Glu190Lys developed higher trypsin levels, particularly at the earlier time points. Interestingly, the slight increase in the initial rate of autoactivation observed with wild-type cationic trypsinogen was absent in the p.Glu190Lys mutant, indicating that CTRC-mediated N-terminal processing of the activation peptide is impaired. Experiments measuring digestion of the Leu81-Glu82 peptide bond in cationic trypsinogen by CTRC demonstrated no difference between wild-type and mutant, confirming that increased autoactivation of the p.Glu190Lys mutant is not due to slower degradation by CTRC (Figure 3C).

### Cellular Secretion of p.Glu190Lys Trypsinogen

**FIGURE 4:**
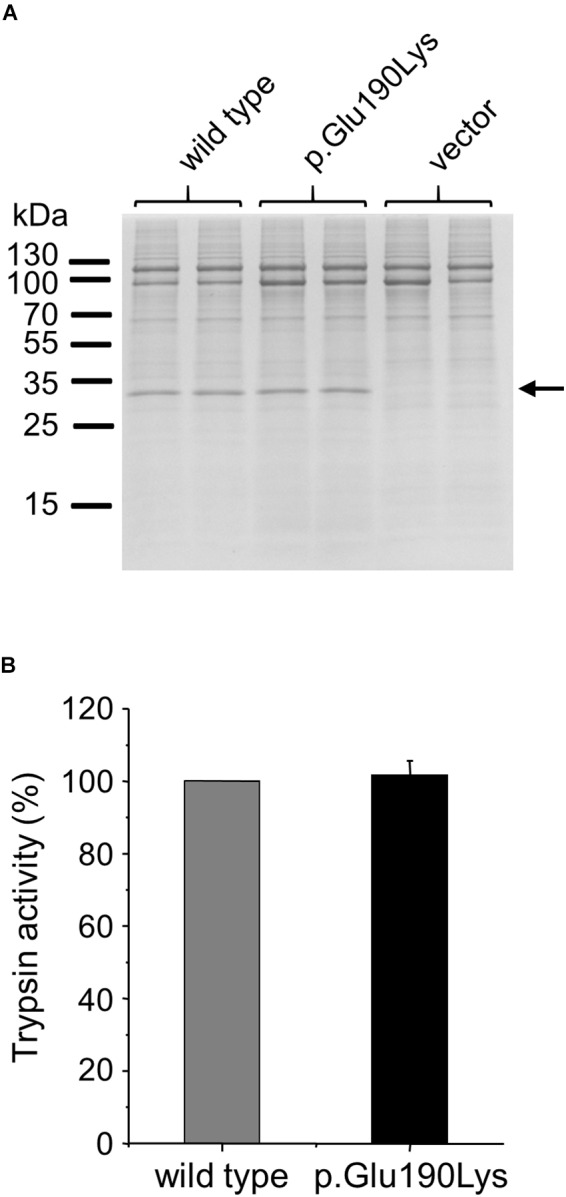
Effect of mutation p.Glu190Lys on the cellular secretion of human cationic trypsinogen. HEK 293T cells were transiently transfected with expression plasmids for wild-type and mutant trypsinogen, and conditioned media were collected after 48 h. Trypsinogen protein levels were determined by SDS-PAGE and Coomassie Blue staining **(A)** and by measuring trypsin activity in the medium after activation with enteropeptidase **(B)**. Trypsin activity was expressed as percent of the wild-type sample (mean ± SD, *n* = 5). Representative gel from three experiments is shown.

We studied the effect of the p.Glu190Lys mutation on secretion of trypsinogen by transiently transfecting HEK 293T cells with wild-type and mutant *PRSS1* expression plasmids. Secretion of cationic trypsinogen in the growth medium was measured by SDS-PAGE and trypsin activity assays after activation of the secreted trypsinogen to trypsin by enteropeptidase (Figure 4). No difference was detected between wild type and mutant, indicating that mutation p.Glu190Lys has no effect on trypsinogen folding and secretion.

## Discussion

With the advent of routine genetic screening of patients with recurrent acute pancreatitis and chronic pancreatitis, findings of rare or private variants in known susceptibility genes has become an increasingly common occurrence. These variants of unknown significance pose a dilemma for the genetic counselor and the clinician and may create unnecessary anxiety for the patient. In the absence of genetic evidence, functional analysis of the variants may shed some light on their clinical significance. If the functional phenotype of a variant matches that of an established risk variant; then there is a high probability for pathogenicity. As a case in point, here we present the rare p.Glu190Lys missense variant in the *PRSS1* gene identified in a Polish subject with chronic pancreatitis. The patient carried no other pathogenic changes in known risk genes such as *CFTR*, *CPA1*, *CTRC*, and *SPINK1*, suggesting a potentially strong pathogenic impact of the novel *PRSS1* mutation. The absence of family history of pancreatitis, however, might indicate that variant p.Glu190Lys has a weaker effect than the archetypal p.Arg122His mutation, which tends to cause disease in multiple generations. We note that caution is warranted with these assumptions as penetrance and expressivity of *PRSS1* mutations can be variable and the single case presented here does not allow firm conclusions. More importantly, the limited genetic data is insufficient to determine pathogenicity of the p.Glu190Lys *PRSS1* variant.

Biochemical analysis successfully resolved this uncertainty and demonstrated that mutation p.Glu190Lys increases autoactivation of human cationic trypsinogen by 2.5-fold in a manner that is independent of CTRC, i.e., the mutation does not reduce CTRC-dependent degradation nor does it increase CTRC-mediated processing of the activation peptide. Catalytic activity of the mutant trypsin was unchanged, indicating that increased autoactivation was likely the result of trypsinogen becoming a better substrate for trypsin. We explain this with the altered surface potential of the mutant trypsinogen, which may facilitate macroscopic attraction between trypsin and trypsinogen or may affect the conformation of the activation peptide. Mutation p.Glu190Lys had no effect on cellular secretion of cationic trypsinogen demonstrating that the misfolding dependent pathway is not involved in the pathogenic mechanism of this variant. Taken together, the functional studies clearly classify mutation p.Glu190Lys into the trypsin-dependent mechanistic group.

In summary, we identified the novel p.Glu190Lys variant in a case of chronic pancreatitis and demonstrated that this mutation increases autoactivation of cationic trypsinogen. We designate p.Glu190Lys as a likely pathogenic variant. The results also caution that analysis of the *PRSS1* gene in patients with suspected genetic predisposition for pancreatitis should include all five exons and exon–intron junctions, so that rare pathogenic variants such as p.Glu190Lys are not overlooked.

## Author Contributions

MS-T and AR conceived and directed the study. AK and AR performed the genotyping. GO and EK provided the patient data. ER performed the structural modeling. ZJ and MS-T designed and analyzed the functional experiments. ZJ performed the experiments. MS-T wrote the manuscript. All authors approved the manuscript.

## Conflict of Interest Statement

The authors declare that the research was conducted in the absence of any commercial or financial relationships that could be construed as a potential conflict of interest.
